# Different spinal subtypes with varying characteristics of lumbar disc degeneration at specific level with age: a study based on an asymptomatic population

**DOI:** 10.1186/s13018-019-1537-7

**Published:** 2020-01-03

**Authors:** Shao-qing Chen, Qing-ping Li, Ying-ying Huang, An-na Guo, Rui-fang Zhang, Pei-pei Ye, Zhi-han Yan, Jia-wei He

**Affiliations:** Department of Radiology, The Second Affiliated Hospital and Yuying Children’s Hospital of Wenzhou Medical University, 109 Xueyuanxi Rd, Wenzhou, 325027 Zhejiang China

**Keywords:** Lumbar, Disc, Degeneration, Sagittal, Alignment, Spine, Subtype, Asymptomatic, Spinopelvic

## Abstract

**Background:**

The relationship between spinal sagittal subtypes and lumbar disc degeneration is unclear. Thus, we aimed to investigate the relationship between lumbar intervertebral disc degeneration and age in asymptomatic healthy individuals with different sagittal alignments.

**Methods:**

In this cross-sectional observational study, we examined 209 asymptomatic young and middle-aged volunteers (123 women and 86 men) who were divided into the following three groups according to age: groups A (20–30 years), B (31–40 years), and C (41–50 years). The volunteers underwent full-spine standing lateral radiography and magnetic resonance imaging (MRI, 3.0 T) of the lumbar spine. Based on panoramic radiography, two observers measured the spinopelvic parameters and classified the spine into Roussouly subtypes. The degree of disc degeneration was assessed based on T2-weighted images according to the Pfirrmann classification.

**Results:**

There was a statistically significant difference in the degree of degeneration of type I spine between groups B and C at L4-L5 (*P* < 0.03) and L5-S1 (*P* < 0.01) and between groups A and C at L1-L2 (*P* < 0.04) and L4-L5 (*P* < 0.01). The degeneration degree of type II spine at all levels were significantly different between groups A and C. No statistically significant difference was found between groups A and B in all subtypes except for type II spine at L1-L2 (*P* < 0.04). A significant difference was found at four levels between groups B and C in type III spine (*P* < 0.05) and between groups A and C. For type IV spine, there was a significant difference in the degree of degeneration at L4-L5 (*P* < 0.02) between groups A and C. Moreover, almost all single parameters were not strongly correlated with the degree of disc degeneration.

**Conclusion:**

The different spinal subtypes have characteristics of lumbar disc degeneration at specific levels with age. We considered that spinal classification could be used as a predictor of lumbar disc degeneration. Our data may be helpful to increase awareness of the relationship between spinal subtypes and lumbar disc degeneration.

**Level of evidence:**

3

## Background

Lumbar intervertebral disc degeneration (LDD), the main cause of lumbar spinal disorders, is associated with biomechanical stresses and increases with age [1–6]. Previous studies have demonstrated that sagittal spinopelvic alignment plays an important role in LDD [7–9]. Therefore, it is meaningful and useful to study the relationship between spinal sagittal morphology and disc degeneration.

The fully upright posture of humans requires coordination of all parts to form different spine sagittal patterns and develop different biomechanical characteristics [10, 11]. Therefore, when investigating the biomechanics of the spine, we tend to consider the spine as a whole. Previous studies that refer to spine classification usually adopted the classification proposed by Roussouly et al. [12] who categorized the spine into four subtypes based on asymptomatic young and middle-aged individuals’ spinopelvic parameters on plain film. On the other hand, magnetic resonance imaging (MRI) is used for an accurate noninvasive evaluation of LDD. Pfirrmann classification is also often adopted to evaluate the degree of the disc degeneration on MRI [13].

Several studies have evaluated the association between spinal sagittal morphology and LDD [9, 14]. Rafael et al. [9], for example, found that Roussouly type II sagittal alignment is significantly associated with early disc degeneration at the L4-L5 level, compared with type IV. However, Torrie et al. [14] proposed that lumbar sagittal subtypes were not statistically significantly correlated with LDD. Therefore, the relationship between spinal sagittal subtypes and LDD has not been clearly explained.

In this study, we hypothesize that the sagittal alignment subtypes may be related to lumbar disc degeneration in asymptomatic healthy individuals.

## Methods

### Sample

After obtaining approval from the local Institutional Review Board, we recruited 209 asymptomatic young and middle-aged volunteers (123 women and 86 men) from our hospital between June 2016 and July 2018. The study objectives were explained to the volunteers, and they provided written informed consent prior to study participation. The inclusion criteria were as follows: (1) age between 20 and 50 years; (2) no history of any spinal surgery; (3) absence of arthropathy in the lower limbs; and (4) no history of neuromuscular disorders. The exclusion criteria were as follows: (1) complaints of back pain, neck pain, or limb numbness caused by degenerative diseases of the spine; (2) heavy manual labor; (3) spinal trauma or tumor; and (4) spinal deformities (including scoliosis, isthmic spondylolisthesis, and irregular end-plate). The participants’ demographic characteristics including body weight, height, and body mass index (BMI) were obtained. According to age, the participants were divided into three groups: group A (ages 20–30 years old), group B (31–40 years old), and group C (41–50 years old).

### Panoramic radiography

**Fig. 1:**
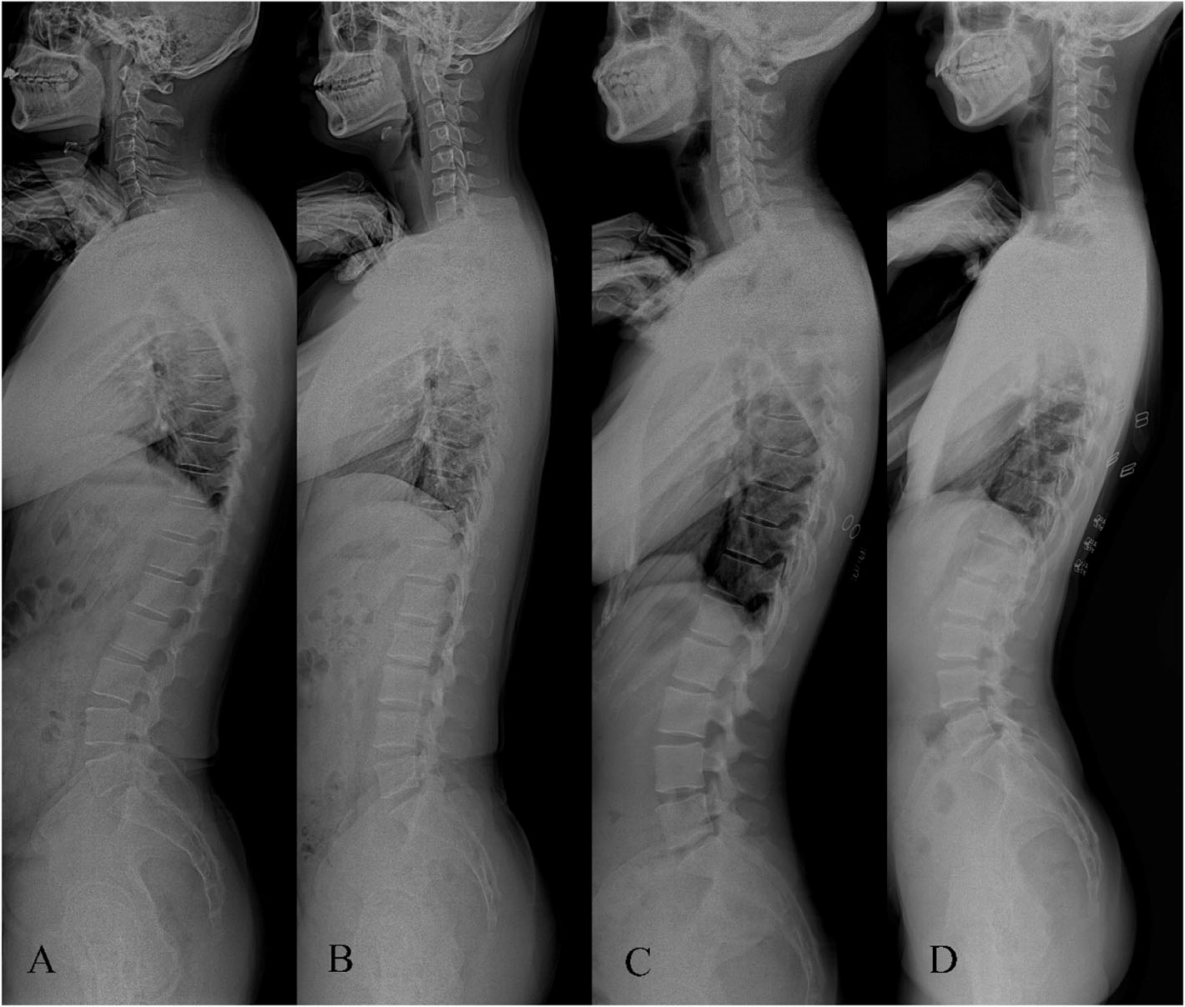
The Roussouly classification I to IV (**a**–**d**)

Full-spine standing lateral radiographs were obtained using the Siemens digital radiography system (SIEMENS YSIO, SIEMENS, Germany) and a picture archiving and communication system (PACS) v3.0 (INFINITT, Shanghai, China). Radiographs were taken in a standard position, wherein participants were asked to stand naturally with both hands on the clavicle with hip and knee joint extension. The radiographs were respectively examined by a spine surgeon and a radiologist, who were qualified for reviewing whole-spine images. The two observers measured the following spinopelvic parameters on PACS: lumbar lordosis (LL), sacral slope (SS), pelvic incidence (PI), pelvic tilt (PT), and sagittal vertical axis (SVA). Based on panoramic radiography, the two observers classified the spine into different subtypes according to the Roussouly classification (Fig. 1). When the SS is less than 35°, the LL arc is nearly absent and the thoracic kyphosis (TK) is larger, and the sagittal alignment is classified as type I. When the distal arch is low and larger and the LL is flat, the sagittal alignment is classified as type II. When the SS is between 35° and 45°, and the TK and LL are harmonious, the sagittal alignment is classified as type III. When the SS is greater than 45°, the LL is larger and the TK is smaller, then the sagittal alignment is classified as type IV. When there is a disagreement about the classification, a third observer’s opinion (a radiologist with over 20 years of experience in musculoskeletal radiology diagnostics) was accepted.

### MRI

To evaluate lumbar intervertebral degeneration, a 3.0-T high-field MRI scanner (Discovery 750, GE Healthcare, US) was used to collect images. T2-weighted images of the lumbar spine in the sagittal plane were acquired using a 2D sequence with the following settings: field of view = 256 × 250 mm; thickness = 5 mm; number of slices = 15; matrix = 256 × 256; echo time = 120 ms; and repetition time = 2500 ms. The two observers assessed the lumbar discs independently, according to the Pfirrmann classification (Table 1). When there is a disagreement about the classification, a third observer’s opinion (a radiologist with over 20 years of experience in musculoskeletal radiology diagnostics) was accepted.

**Table 1:** Disc degeneration grading according to Pfirrmann et al.

Grade	Structure	Distinction of nucleus and anulus	Signal intensity	Intervertebral disc height
I	Homogeneous, bright white	Clear	Hyperintense, isointense to cerebrospinal fluid	Normal
II	Heterogeneous with or without horizontal bands	Clear	Hyperintense, isointense to cerebrospinal fluid	Normal
III	Heterogeneous, gray	Unclear	Intermediate	Normal to slightly decreased
IV	Heterogeneous, gray to black	Lost	Intermediate to hypointense	Normal to moderately decreased
V	Heterogeneous, black	Lost	Hypointense	Collapsed disc space

### Statistical analyses

The SPSS software (ver. 19.0; SPSS Inc., Chicago, IL, USA) was used to conduct all analyses. Demographic data conforming to a normal distribution were expressed as means ± standard deviation. Differences in demographic characteristics among the three groups were examined for statistical significance using Student’s *t* test. The Mann-Whitney *U* test was used to compare the degree of lumbar disc degeneration among the three age groups (differences with *P* < 0.05 were considered statistically significant). Spearman’s correlation was used to verify the correlations between the spinopelvic parameters and disc degeneration degree. The interobserver assessment of spinopelvic variables adopted the intraclass correlation coefficient (ICC) and the weighted Kappa coefficient for Roussouly and Pfirrmann classifications.

## Results

The mean values and the standard deviations of the demographic data are summarized in Table 2. There is no significant difference among the three age groups in height, weight, and BMI. The spinal subtypes of the participants were as follows: type I (*n* = 37), type II (*n* = 67), type III (*n* = 77), and type IV (*n* = 28).

**Table 2:** General date between three age groups

	A	B	C
Male/Female	30/34	32/38	24/51
ages	25.74 ± 2.14	35.40 ± 2.70	46.42 ± 3.14
Height	1.66 ± 0.09	1.65 ± 0.08	1.64 ± 0.07
Weight	62.90 ± 9.69	59.89 ± 10.39	61.62 ± 11.31
BMI	22.86 ± 2.87	21.93 ± 2.64	22.93 ± 3.24

*BMI* body mass index

Values are expressed as mean ± SD

Statistical analysis of clinical date in each age group

Table 3 shows the difference in the degree of degeneration at each disc level on MRI among the three age groups. We found significant differences at all disc levels between groups B and C and between groups A and C. However, no significant difference was observed between groups A and B at each disc level.

**Table 3:** Difference between the age groups on the degeneration grades at each disc level

Age Groups	L1-L2		L2-L3		L3-L4		L4-L5		L5-S1	
	u	*P*	u	*P*	u	*P*	u	*P*	u	*P*
A vs B	− 1.72	0.08	− 1.59	0.11	− 1.83	0.67	− 1.81	0.07	− 0.99	0.33
B vs C	− 4.18	0.00*	− 2.88	0.00*	− 4.23	0.00*	− 4.48	0.00*	− 3.39	0.00*
A vs C	− 5.05	0.00*	− 3.65	0.00*	− 5.39	0.00*	− 6.03	0.00*	− 4.19	0.00*

* There was a statistically significant difference (*P* < 0.05)

Table 4 shows the correlations (r) between the sagittal spinopelvic parameters and the degree of degeneration at each disc level among the different age groups. There were slightly negative correlations between the LL and disc degeneration grade at L4-L5 in groups B (*r* = − 0.26) and C (*r* = − 0.25). Moreover, the other parameters were not significantly correlated with intervertebral degeneration.

**Table 4:** Spearman’s correlation (*r*) between the spinopelvic parameters and the Pfirrmann disc degeneration degree at each lumbar disc level

	A					B					C				
	L1-L2	L2-L3	L3-L4	L4-L5	L5-S1	L1-L2	L2-L3	L3-L4	L4-L5	L5-S1	L1-L2	L2-L3	L3-L4	L4-L5	L5-S1
LL	0.04	− 0.04	− 0.08	0.12	− 0.04	0.04	0.03	− 0.23	− 0.25*	− 0.18	− 0.01	− 0.09	0.17	− 0.26*	0.04
SS	0.17	0.14	− 0.07	− 0.12	0.05	− 0.05	− 0.02	0.14	0.21	0.14	− 0.09	− 0.05	− 0.18	−0.16	0.06
PT	− 0.09	0.00	0.16	− 0.16	− 0.07	0.23	0.07	0.01	0.05	0.04	0.19	− 0.03	0.04	− 0.01	− 0.05
PI	− 0.03	0.14	0.02	− 0.11	0.09	0.11	− 0.01	0.08	0.19	0.11	− 0.02	− 0.01	− 0.11	− 0.18	0.09
SVA	0.05	− 0.07	− 0.01	0.08	− 0.15	0.11	− 0.04	0.10	− 0.08	0.08	− 0.10	− 0.04	0.14	− 0.12	− 0.06

*There was a statistically significant correlation (*P* < 0.05)

*LL* lumbar lordosis, *SS* sacral slope, *PT* pelvic tilt, *PI* pelvic incidence, *SVA* sagittal vertical axis

When we compared the disc degeneration grade among the three groups at each disc level (Table 5), no statistically significant difference was found between groups A and B in all subtypes except for type II spine at L1-L2 (*P* < 0.04).

**Table 5:** Difference between the age groups on degeneration degree in four Roussouly types

Roussouly types	L1-L2						L2-L3						L3-L4						L4-L5						L5-S1					
	A vs. B		B vs. C		A vs. C		A vs. B		B vs. C		A vs. C		A vs. B		B vs. C		A vs. C		A vs. B		B vs. C		A vs. C		A vs. B		B vs. C		A vs. C	
	u	*P*	u	*P*	u	*P*	u	*P*	u	*P*	u	*P*	u	*P*	u	*P*	u	*P*	u	*P*	u	*P*	u	*P*	u	*P*	u	*P*	u	*P*
Type I	− 0.45	0.66	− 1.84	0.07	− 2.07	0.04*	− 0.82	0.41	− 0.30	0.77	− 0.99	0.32	− 1.18	0.24	− 1.14	0.26	− 1.88	0.06	− 1.36	0.17	− 2.21	0.03*	− 2.88	0.00*	− 0.37	0.71	− 2.50	0.01*	− 1.66	0.10
Type II	− 2.04	0.04*	− 2.42	0.02*	− 3.43	0.00*	− 0.85	0.40	− 2.38	0.02*	− 2.47	0.01*	− 1.76	0.08	− 1.38	0.17	− 2.68	0.01*	− 0.95	0.34	− 1.20	0.23	− 2.20	0.03*	− 0.92	0.36	− 1.38	0.17	− 2.16	0.03*
Type III	− 0.51	0.61	− 2.59	0.01*	− 2.80	0.01*	− 0.66	0.51	− 1.37	0.17	− 1.93	0.05	− 0.21	0.84	− 3.96	0.00*	− 4.52	0.00*	− 0.51	0.61	− 3.47	0.00*	− 4.11	0.00*	− 0.56	0.57	− 1.98	0.04*	− 2.71	0.01*
Type IV	− 0.20	0.84	− 1.03	0.30	− 1.13	0.26	− 1.10	0.27	− 1.41	0.16	− 1.41	0.16	− 0.13	0.90	− 0.52	0.61	− 0.38	0.71	− 0.91	0.36	− 1.87	0.06	− 2.39	0.02*	− 0.27	0.79	− 1.19	0.23	− 1.33	0.18

* There was a statistically significant difference (*P* < 0.05)

There was a statistically significant difference in the degree of degeneration of type I spine between groups B and C at L4-L5 (*P* < 0.03) and L5-S1 (*P* < 0.01) and between groups A and C at L1-L2 (*P* < 0.04) and L4-L5 (*P* < 0.01). The disc degeneration grade of type II spine was significantly different at all spinal levels between groups A and C. Comparing group B with group C, there was a statistically significant difference in the degree of degeneration of type II spine at L1-L2 (*P* < 0.02) and L2-L3 (*P* < 0.02).

Significant differences in the degree of degeneration of type III spine were found at the four disc levels between groups B and C (*P* < 0.05) and between groups A and C. Moreover, there was a significant difference in the degree of degeneration of type IV at L4-L5 (*P* < 0.02) between groups A and C.

The Kappa coefficient for Roussouly and Pfirrmann classifications were 0.76 (95% CI = 0.69–0.83) and 0.83 (95% CI = 0.79–0.86), respectively. The ICCs for all spinopelvic parameters were excellent (LL: 0.95, 95% CI = 0.92–0.96; SS: 0.85, 95% CI = 0.78–0.90; PT: 0.80, 95% CI = 0.75–0.85; PI: 0.84, 95% CI = 0.79–0.89; SVA: 0.90, 95% CI = 0.87–0.92).

## Discussion

In this study, the spinal alignment of the asymptomatic young and middle-aged individuals enrolled was classified into four spinal subtypes using X-ray according to the Roussouly classification. We analyzed the disc degeneration using MRI among the different age groups and found that the disc levels with increased degree of degeneration were different for each spinal subtype.

The sagittal alignment and curvature of the spine have been considered as important indicators of disc load and pressure in many studies [7, 8, 15, 16]. However, most researches focused on the patients with lumbar disc disorders or clinical symptoms, and studies investigated the effect of sagittal alignment on disc degeneration in asymptomatic young and middle-aged individuals are few. Thus, we explored the relationship between disc degeneration and spinal subtypes in asymptomatic people without the influence of lumbar diseases.

In our study, we found that the degree of degeneration for type I was accelerated mainly at L4-L5 (Table 5). In type I spine, the thoracic kyphosis is large, the lumbar lordosis is small, and the lumbar curvature is mainly in the distal lumbar spine. Thus, we considered that the pressure on the distal lumbar intervertebral disc is higher and prone to degenerate. Roussouly et al. [12] suggested that there was a high prevalence of degeneration at L4-S1 for type I, and hyperextension at L4-S1 may induce a nutcracker L5 spondylolysis [17]. This finding was similar to our results.

Among the three age groups, the intervertebral disc degeneration of type II was concentrated at L1-L2 and L2-L3 (Table 5), which was remarkably different from type I. We suspected that it may be because the sagittal curvature of type II is mild, especially at the junction of the thoracic and lumbar vertebrae. As a connecting part, the upper lumbar intervertebral disc bears a heavy load, and given the shape of the spine, the pressure cannot be dispersed by the posterior disc and facet joints well; thus, the lumbar disc is more likely to degenerate with age [15].

In type III spine, except for L2-L3, the degeneration was aggravated at all levels among the three age groups. We believed that this was due to the fact that the thoracic kyphosis and lumbar lordosis of type III spines are harmonious, resulting in an average pressure on the lumbar area. Therefore, lumbar degeneration is common without specific degeneration. Comparable to our results, Pinheiro et al. found that the type III spine has an average shape without characteristics of any degeneration [17].

Notably, a significant difference in the degree of degeneration of type IV spine was only found at L4-L5 between groups A and C. Type IV is characterized by small thoracic and great lumbar curvature. Pinheiro et al. demonstrated that, if the lumbar curvature is prominent, forces are distributed well between the disc (anterior) and the facet joints (posterior) [17]. Previous studies have found that individuals with type IV spine have the lowest incidence of low back pain. On the other hand, when individuals maintain the lordosis curvature, posterior arthritis may occur and degenerative spondylolisthesis may later develop at L4-L5 [17]. Our research was consistent with these views.

In addition, we found that the type II spine was the only subtype that had significant degenerative changes in all the five intervertebral disc levels when comparing groups A and C. Moreover, it was the only subtype that had significant disc degeneration when comparing groups A and B. The sagittal shape of type II spine is almost flat, which is referred to as “flat back.” When the lumbar spine is hypolordotic and flat, contact forces cannot be distributed well and primarily act on the anterior region of the spine, vertebra, and intervertebral discs, thereby increasing disc pressure [15–18].

Another clinically relevant finding of our study was that there were pervasive differences between groups B and C, but rare significant differences were found between groups A and B (Table 3). Cheung et al. discovered that disc degeneration occurs in 42% of individuals younger than 30 years old, and the frequency of disc degeneration increased with age (30–40 years: 48%; 40–49 years: 70%; and > 55 years: 88%) [1]. There was a slow increase in the disc degeneration in younger patients than in those aged 30 years to 30–40 years, which was consistent with our results. Besides, we could see a dramatic increase in the degree of degeneration in their 40s, indicating that the peak of increased prevalence of disc degeneration may be from approximately 40 years of age. We need to pay attention to this to reduce the incidence of disc degeneration.

In 2016, Rafael et al. identified moderate associations between the spinopelvic parameters and disc degeneration in type II spine among asymptomatic people [9]. In our study, we only identified slightly negative correlations between the LL and disc degeneration grade at L4-L5 in groups B and C (Table 4). Although the results were different, they also indicated that a single parameter could not serve as a stable predictor for the disc degeneration. According to the abovementioned results, we thought that intervertebral disc degeneration was associated with the whole spinal curvature and morphology.

Therefore, we considered that the classification of the spine is a more reasonable and reliable method to predict the disc degeneration. Morphological predictors of altered disc load outcomes were sagittal balance parameters in the thoracic spine and anatomic angles in the lumbar spine [19]. As expected, the Roussouly classification for categorizing the spinal alignments is inclusive and indicative, which considered most of the pelvic and spinal parameters [14].

The Roussouly classification is based on the spinal characteristics of normal asymptomatic young adults; hence, each classification is considered normal. However, a number of studies have shown that type II spines are more prone to lumbar disc degeneration or clinical symptoms such as low back pain [7, 17]. Moreover, the other three subtypes have their own high incidence of level-specific discopathies [17]. In our study, we also found that the four Roussouly subtypes had different characteristics of disc degeneration. Therefore, we proposed that targeted studies should be carried out for different types of spine including their clinical treatment. In addition, our results may be related to the clinical diagnosis and treatment, especially to the recent attractive biological treatments. Biologic treatments, such as percutaneously injected multipotent mesenchymal stem cell (MSC) therapy, involve administration of biologic factors into the intervertebral disc to enhance matrix synthesis, delay degeneration, or impede inflammation [20–22]. If we could figure out the regularity of disc degeneration in different subtypes and predict it, then we could identify preventive measures and conduct early biotherapy targeted for each subtype.

However, several limitations still exist in our study. First, because of the nonuniform population distribution of the Chinese participants, the sample size for type IV spine is small. Second, the purpose of our study was to explore whether the curvature of the spine could affect the degeneration of intervertebral disc in asymptomatic people. Some studies indicated that heavy work could accelerate disc degeneration [4, 6]. This may affect our results; thus, we excluded individuals engaged in performing heavy work. Therefore, our findings may not apply to heavy manual workers. Moreover, although we excluded heavy manual laborers, our study still lacked some clinical data such as smoking, drinking, and so on. In addition, we recruited young and middle-aged people to minimize the effects of spinal parameters with age. However, it still affected our results more or less, which was inevitable.

## Conclusion

We found that different spinal subtypes have different characteristics of lumbar disc degeneration at specific levels with age. We considered that spinal classification could be used as a predictor of lumbar disc degeneration. Our results may be helpful to increase researchers’ and doctors’ awareness of the relationship between spinal subtypes and lumbar disc degeneration, thus could play a certain role in clinical diagnosis and treatment of lumbar spine disorders related to disc degeneration.

## Data Availability

All data and materials were in full compliance with the journal’s policy.

## Abbreviations

LDD: Lumbar intervertebral disc degeneration
LL: Lumbar lordosis
MRI: Magnetic resonance imaging
PI: Pelvic incidence
PT: Pelvic tilt
SS: Sacral slope
SVA: Sagittal vertical axis
TK: Thoracic kyphosis
